# RNA Interference Therapeutics for Hereditary Amyloidosis: A Narrative Review of Clinical Trial Outcomes and Future Directions

**DOI:** 10.7759/cureus.62981

**Published:** 2024-06-23

**Authors:** Prashil Dave, Puneet Anand, Azra Kothawala, Prakhyath Srikaram, Dipsa Shastri, Anwar Uddin, Jill Bhavsar, Andrew Winer

**Affiliations:** 1 Internal Medicine, State University of New York Downstate Health Sciences University, New York, USA; 2 Pediatrics, Icahn School of Medicine at Mount Sinai/Elmhurst Hospital Center, New York, USA; 3 Medicine, Jawaharlal Nehru Medical College, Ahmedabad, IND; 4 Internal Medicine, Baptist Memorial Hospital, Oxford, USA; 5 Internal Medicine, East Tennessee State University (ETSU), Johnson City, USA; 6 Internal Medicine, Medical College Baroda, Baroda, IND; 7 Urology, State University of New York Downstate Health Sciences University, New York, USA

**Keywords:** amyloid transthyretin, management of hereditary amyloidosis, amyloidosis treatment, rna interference therapeutic, transthyretin amyloidosis, hereditary transthyretin amyloidosis

## Abstract

Hereditary transthyretin amyloidosis (ATTR) is an autosomal dominant, life-threatening genetic disorder caused by a single-nucleotide variant in the transthyretin gene. This mutation leads to the misfolding and deposition of amyloid in various body organs. Both mutant and wild-type transthyretin contribute to the resulting polyneuropathy and cardiomyopathy, leading to significant sensorimotor disturbances and severe cardiac conditions such as heart failure and arrhythmias, thereby impacting quality of life. Despite several treatments, including orthotopic liver transplantation and transthyretin tetramer stabilizers, their limitations persisted until the introduction of RNA interference (RNAi). RNAi, a means to regulate mRNA stability and translation of targeted genes, has brought about significant changes in treatment strategies for ATTR with the introduction of patisiran in 2018. This study reviews patisiran, vutrisiran, inotersen, and eplontersen, developed for the treatment of ATTR. It provides an overview of the clinical trial outcomes, focusing mainly on quality of life, adverse reactions, and the future of RNAi-based therapies.

## Introduction and background

Hereditary transthyretin amyloidosis (ATTR) is a rare, autosomal dominant disorder characterized by the pathological deposition of amyloid in various organ systems [1]. This extracellular accumulation of transthyretin-derived amyloid fibrils can lead to various pathological conditions, primarily cardiomyopathy and polyneuropathy [1]. The clinical manifestations largely depend on the specific genetic variant [1]. The most common point mutation associated with ATTR polyneuropathy is Val30Met, while others are linked with cardiomyopathy or a mixture of both [1]. ATTR affects both central and peripheral nerves [2]. CNS manifestations include leptomeningeal amyloidosis and spinal canal stenosis, while peripheral nervous system (PNS) manifestations include polyneuropathy, carpal tunnel syndrome, and autonomic dysfunction [2]. A study by Koike H et al. suggests that even with the same genetic mutation, disease presentation can vary significantly [3]. For instance, low endemic foci are associated with late-onset, predominantly sensorimotor clinical features, whereas high endemic foci are associated with early onset and severe autonomic dysfunction [3]. New therapeutic strategies using antisense oligonucleotides (ASOs) and small interfering RNA (siRNA) show promise in interrupting amyloid production in both variant and wild-type TTR forms [4].

RNAi is an RNA-mediated gene silencing process that primarily uses siRNA and microRNA (miRNA) through the RNA-inducing silencing complex or RISC, affecting mRNA translation [5]. Due to the base pairing phenomenon, this silencing is highly specific to the targeted genes [5]. ASOs are synthetic messenger RNA (mRNA) that binds to target mRNA through complementary base pairing [6]. However, for effective systemic circulation, tissue penetration, and uptake, RNAi must undergo various modifications due to its inherent molecular structure and target tissue environment [6]. Several strategies were developed, including lipid-based nanoparticles, polymer-based nanoparticles, and chemical modification [7]. Many clinical trials were conducted with selected RNAi-based therapies, yielding positive results in hypercholesterolemia, acute intermittent porphyria, hepatitis B, hepatocellular carcinoma, and alpha-one antitrypsin deficiency [8]. In the context of ATTR, emerging therapies aim to reduce the production of amyloidogenic proteins by decreasing TTR synthesis, stabilizing TTR tetramers, and disrupting amyloid fibrils [9]. There has been significant progress in treating ATTR, moving from purely symptomatic treatment to the application of RNAi. RNAi is highly effective and significantly reduces the production of both wild-type and variant forms in the liver [9]. Four FDA-approved RNAi medications for ATTR polyneuropathy are patisiran, vutrisiran, inotersen, and eplontersen. Overall data demonstrate the safety and efficacy of these RNAi medications; however, a thorough understanding of each drug is essential for appropriate clinical application. This review outlines the safety, effectiveness, and quality of life outcomes of these treatments.

## Review

An overview of RNAi

RNA interference, or RNA silencing, is a contemporary form of 'transcript-targeted therapy' that functions at the molecular level, modifying the levels of both coding and non-coding RNAs, either transcriptionally or post-transcriptionally, to provide therapeutic benefits [10]. RNAi typically involves the degradation of double-stranded RNA (dsRNA) into siRNAs, which, in turn, degrade targeted mRNA molecules, resulting in the downregulation of gene expression [11]. This mechanism was discovered in 1998 by Andrew Fire and Craig Mello, a breakthrough that earned them the Nobel Prize in Physiology or Medicine in 2006 [11]. Additionally, siRNAs are relatively straightforward to synthesize, even on a large scale, which can be a significant challenge with biologicals [12]. Since siRNAs operate at the post-transcriptional level, acting on mRNA rather than protein, they can target and potentially inhibit the action of genes that are otherwise deemed undruggable, for which no protein inhibitors exist or cannot be obtained [13]. The potential applications of RNAi have increased significantly, and it is now being evaluated as a therapeutic tool for various diseases, such as cancers, viral infections, and neurodegenerative disorders [14].

The siRNAs are composed of guide and passenger strands [15]. Within the cell's cytoplasm, the guide strand of the siRNAs is incorporated into the RISC, targeting the transcript with total complementarity [16]. This triggers an endonucleolytic cleavage, typically mediated by a RISC-associated protein known as 'Argonaute-2' or 'Ago2' [17]. This results in the targeted mRNA's degradation and halts protein translation [17]. The passenger strand is usually discarded after degradation [18]. Two small RNA molecules usually act as the initiators of the RNAi pathway: endogenous microRNA, produced by the cell's genome, and exogenous small interfering RNA, derived from an extracellular genome [19]. Notably, miRNAs can recognize mRNA targets with imperfect complementarity and repress translation, thus silencing the genes. Unlike other oligonucleotide systems, RNAi operates on a catalytic mechanism, necessitating lower nucleic acid delivery to the cell. Furthermore, siRNA-based cleavage is more efficient than ribozymes [19]. Highly potent siRNAs show activity even at picomolar concentrations, and the delivery of fewer than 2000 siRNA molecules per cell is sufficient to achieve specific gene knockdown [20].

Amyloid Deposition in ATTR

ATTR represents a rare, rapidly progressive, and fatal autosomal dominant disorder associated with mutations in the TTR gene [21]. This disorder is characterized by the extracellular deposition of amyloid, leading to the deterioration of various organ systems and subsequent impairment of functionality. The peripheral nerves, heart, kidney, eye, and GI tract are most commonly affected [21]. Systemic amyloid deposition can be attributed to either wild-type or variant amyloidogenic TTR (ATTRwt and ATTRv, respectively) [22]. ATTR Amyloidosis can be classified as a gain-of-function toxic protein misfolding disease [23]. In this disease, the variant TTR assembles into amyloid fibrils in the extracellular space, impairing organ function [23]. While TTR is synthesized at various sites, most of its production occurs in the liver [22]. The liver-synthesized TTR is primarily responsible for most ATTR manifestations, such as neuropathy and cardiomyopathy [22].

The natural progression of ATTR typically results in severe disability, heart failure, and mortality within 4-15 years from onset, with the specific timeline subject to genotype variation [24]. However, the advent of innovative therapeutic strategies has transformed the course of this disease [24]. These therapeutic strategies focus on TTR production, stabilization, and amyloid deposit removal [25]. Disease-modifying therapies have shown significant efficacy when implemented early in the disease progression, emphasizing the necessity of prompt diagnosis [26]. The introduction of these innovative therapeutic approaches marks a new phase in ATTR management, offering enhanced patient outcomes and quality of life [26].

Patisiran

An Overview of Patisiran 

Patisiran was one of the first siRNAs licensed with an N-acetylgalactosamine (GalNAc) linkage, facilitating effective liver-directed delivery where most TTR protein is synthesized [27, 28]. This double-stranded siRNA oligonucleotide is 21 bases long [29]. It binds to a genetically preserved sequence in the 3’ untranslated region (3’UTR) of both wild-type and mutant TTR mRNA. With the help of RISC and RNAi mechanisms, TTR mRNA is degraded, reducing TTR protein levels in both serum and tissue [29, 30]. Studies indicate that a single intravenous administration of patisiran reduces the mean serum TTR by 80% within 2 weeks, with consistent results across varying patient genotypes, genders, ages, and races [29]. Potential side-effects of patisiran, mainly related to infusion, can be mitigated with premedication using a corticosteroid and antihistamine administered intravenously, along with oral acetaminophen, given at least 60 minutes before infusion [30]. Other common adverse reactions include upper respiratory tract infections, infusion-related myalgia, flushing, nausea, rash, and blood pressure fluctuations. While patisiran has no absolute contraindications, its safety and efficacy in children, pregnant women, and those with severe liver or kidney dysfunction are not yet well established. Mild to moderate renal impairment or mild hepatic dysfunction does not impact patisiran exposure or TTR reduction [29].

Outcomes of Patisiran-Based Clinical Trials 

In phase-1 testing (NCT01148953 and NCT01559077), two intravenously infused siRNA formulations were studied, ALN-TTR01 and ALN-TTR02 (later named patisiran). Patisiran demonstrated significant knockdown rates until day 28 for those receiving doses of 0.15-0.5 mg/kg and at 0.3 mg/kg, achieving over 50% TTR lowering by day 3. At dosages of 0.15 and 0.3 mg/kg, nadirs of 82.3% and 86.8% TTR lowering were observed between days 10 and 15 [27]. The phase-2 clinical trial (NCT01617967) involved adults with biopsy-proven ATTRv and mild-to-moderate polyneuropathy [31]. Excluded were those with prior liver transplants, unstable angina or myocardial infarction within the past six months, New York Heart Association (NYHA) class III or IV heart failure, pregnancy, or other systemic medical conditions. Of the 29 patients enrolled, 26 completed the study. TTR levels reached nadirs of 83.8% and 86.7%, respectively. Serial TTR protein levels were reduced in a dose-dependent manner across cohorts. The mean area under the curve (AUC) and maximal plasma concentration increased proportionally to the dose after both doses. Patisiran demonstrated suppression of both wild-type and mutated TTR in patients with the p.Val50Met mutation [31].

The Phase III APOLLO trial (NCT01960348) included patients aged 18-85 years with a diagnosis of ATTRv amyloidosis, a neuropathy impairment score (NIS) of 5-130, a polyneuropathy disability (PND) score ≤ IIIb, and satisfactory renal and liver function [31]. Exclusions encompassed prior or planned liver transplants, neuropathy unrelated to ATTRv amyloidosis, diabetes types 1 and 2, active hepatitis B or C infection, HIV, and use of specific medications without a washout period [30]. In total, 93% of patients in the treatment group and 71% in the placebo group successfully finished the trial. At 18 months, the patisiran group experienced a least squares mean difference of −34 points in the mNIS+7 score (primary outcome), with differences becoming apparent by nine months. Additionally, the patisiran group experienced less decline in quality of life at 18 months as measured by the Norfolk Quality of Life-Diabetic Neuropathy questionnaire, with a least squares mean difference between groups of -21.1 points, p < 0.001. Secondary efficacy measures included the Norfolk Quality of Life-Diabetic Neuropathy and COMPASS-31 scale, showing significant improvements in those treated with patisiran compared to the placebo at 18 months. Adverse events were reported in 97% of patients, with a similar frequency of serious events between the patisiran and placebo groups [30]. Table 1 highlights the three major patisiran-based clinical trials.

**Table 1:** Clinical trials based on patisiran. N: Number of participants; CT: Clinical trial; TTR: Transthyretin; IRR: Infusion-related reaction; RBP: Retinol-binding protein; RCT: Randomized controlled trial; mNIS: Modified neuropathy impairment score; QOL: Quality of life; QOL-DN: Quality of life diabetic neuropathy; ITT: Intention to treat; CFT: Cardiac function test; pBNP: Pro-brain natriuretic peptide.

Trial ID	Study design	Intervention	N	Primary outcome	Secondary outcome	Status of study
NCT02510261 [11]	Phase III CT	Patisiran infusion was given to patients from APOLLO and phase 2 OLE groups.	211	Sustained TTR reductions and continued improvement in mNIS +7 scores.	Improvement in Norfolk QOL-DN, COMPASS-31, and autonomic functions in the APOLLO- Patisiran group. Positive effects on CFT and stabilized pBNP levels.	Complete
NCT01559077 [27]	Phase 1 CT	ALN-TTR01 was given at doses 0.01 to 1 mg/ kg. ALN-TTR02 was given at doses 0.01- 0.5 mg/ kg	49	Rapid, dose-dependent lowering of transthyretin levels was seen.	IRR in ALN-TTR01 and ALN-TTR02 were 20.8% and 7.7%, respectively.	Complete
NCT01960348 [30]	Phase III CT	Patisiran or placebo every three weeks.	225	Less worsening in the mNIS +7 scores in the patients treated with patisiran.	Better QOL on Norfolk QOL-DN. Gait speed improvement in a 10-minute walk. Better cardiac structure and function.	Discontinued
NCT01617967 [31]	Phase II CT	Patisiran: 0.01, 0.05, 0.15 or 0.3 mg/kg every four weeks; or 0.3 mg/ kg every three weeks.	29	Patisiran effectively reduced both mutant and wild type-TTR levels.	Serum TTR knockdown is directly related to RBP and Vitamin A levels.	Complete

Participants from the phase II and III trials were invited to a Global Open-Label Extension Study (NCT02510261), where they received 0.3 mg/kg of patisiran via IV infusion every three weeks for five years. A 12-month interim analysis showed improvement in mNIS+7 scores. Improvements were also observed in Norfolk QOL-DN, COMPASS-31, and autonomic function in the APOLLO-patisiran group. Positive cardiac function effects were observed, with NT-proBNP levels remaining stable in patisiran-treated patients and improving in the APOLLO-placebo group upon Patisiran initiation (Table 1) [11].

Vutrisiran

Outcomes of Clinical Trials Based on Vutrisiran

In phase I, a randomized, single-blind, placebo-controlled study, the pharmacodynamics, pharmacokinetics, and safety of Vutrisiran (5-300 mg) were evaluated in 80 healthy subjects [32]. Vutrisiran demonstrated potent and sustained TTR reduction in a dose-dependent manner, with a mean maximum TTR reduction of 57-97%, maintained for over 90 days. Rapid absorption was noted (peak plasma concentration at 3-5 hours post-dose), a short plasma half-life (4.2-7.5 hours), and a dose-proportional increase in plasma concentrations. Vutrisiran's safety profile was acceptable, with the most common treatment-related adverse event being mild, transient injection site reactions in 6.7% of subjects [32-38].

In phase 3, the HELIOS-A clinical trial (NCT03759379), the primary endpoint was the change in modified NIS + 7 (mNIS+7) at month 9, compared to the placebo group of the APOLLO study used as an external control [12]. Secondary endpoints included the Norfolk QOL-DN, gait speed, mNIS+7, 10-MWT, nutritional status, disability, and non-inferiority in serum TTR level percent reduction. Vutrisiran treatment significantly improved mNIS+7 at month 9 (-2.24 in vutrisiran and +14.76 in placebo) and month 18 (48.3% of patients in the vutrisiran group showed improvement in mNIS+7 versus 3.9% in the external placebo group). Moreover, significant improvements were observed in the Norfolk QOL-DN score at Month 9 (LS mean change from baseline: -3.3 for vutrisiran and +12.9 for placebo) and 18 (−1.2 in the vutrisiran group and +19.8 in the placebo group). All other secondary endpoints showed significant improvements with vutrisiran treatment [12]. Table 2 highlights the major clinical trials based on vutrisiran, inotersen, and eplontersen.

**Table 2:** Clinical trials on vutrisiran, inotersen, and eplontersen. IV: Intravenous; N: Number of participants; CT: Clinical trial; TTR: Transthyretin; SC: Subcutaneously; RBP4: Retinol-binding protein 4; QoL: Quality of life; QoL-DN: Quality of life-diabetic neuropathy; MAE: Major adverse event; GN: Glomerulonephritis; TCP: Thrombocytopenia.

Trial ID	Study design	Intervention	N	Primary outcome	Secondary outcome	Status of study
NCT03759379 [12]	Phase III CT	Vutrisiran 25 mg SC every three months or Patisiran 0.3 mg/kg IV every three weeks.	164	Statistically significant improvement in mNIS +7 score.	Significant improvement in the Norfolk QOL-DN score.	Active but not recruiting
NCT04136184 [14]	Phase III CT	Eplontersen SC every four weeks, reference group received SC Inotersen and SC placebo weekly.	168	Reductions in serum transthyretin. Lesser mean change from baseline for mNIS +7 composite score and Norfolk QoL-DN.	Adjusted mean change from baseline to week 35 in neuropathy symptom and change total score was 0.8 in the Eplontersen group.	Complete
NCT02797847 [32]	Phase I CT	Vutrisiran	80	Reduction in serum TTR.	Safety profile found to be acceptable.	Complete
NCT01737398 [37]	Phase III CT	Inotersen SC 300 mg once weekly.	172	Significantly fewer changes from baseline to week 66 for mNIS +7 and Norfolk QOL-DN score.	MAE in the Inotersen group included GN and TCP.	Complete
NCT02175004 [37]	Open-label extension study of NEURO-TTR trial	Inotersen SC injection for up to five years.	222	Continued efficacy after three years, with no additional safety concerns or signs of increased toxicity reported.	Extended treatment with Inotersen slowed the progression of polyneuropathy and maintained quality of life.	Complete
NCT03728634 [38]	Phase I CT	Eplontersen multiple dose cohorts	47	No serious adverse events were noted in the study.	Reduced TTR levels from baseline. Dose-dependent prolonged reductions in RBP4.	Complete

Inotersen

Outcomes of Inotersen-Based Clinical Trials

The NEURO-TTR trial, a phase II/III, 15-month, randomized, double-blind, placebo-controlled study, examined the safety and efficacy of inotersen in patients with hATTR amyloidosis with polyneuropathy. It included adults aged 18 to 82 years with a NIS of 10 to 130, documented TTR mutation, and amyloid deposits on biopsy [33, 34]. The results demonstrated that Inotersen significantly improved both neurological function (least squares mean difference -19.7 points, 95% CI -26.4 to -13.0; P<0.001 for mNIS+7) and quality of life measures (least squares mean difference -11.7 points, 95% CI -18.3 to -5.1; P<0.001 for Norfolk QOL-DN) compared to placebo [13]. Notably, Inotersen was first evaluated in a phase 1, randomized, placebo-controlled, double-blind, dose-escalation study (IONIS-TTRRx) involving healthy volunteers aged 18 to 55 [35]. A dose-dependent reduction in serum RBP4 levels was also observed at all doses. Most adverse events (95%) reported were mild and included somnolence (33%), headache (18%), and increases in CRP and blood CPK levels [35]. In the open-label extension (OLE) study, most patients (97%) who completed the NEURO-TTR trial showed sustained benefits in neurological and quality-of-life measures after two years of Inotersen treatment (Table 2) [13, 36].

Eplonterson

*Outcomes on Clinical Trials of hATTR Treatment With Eplonterson* 

A randomized, placebo-controlled, phase 1 study evaluated eplontersen (AKCEA-TTR-LRx) in healthy volunteers (NCT03728634). The primary endpoint was safety and tolerability, with pharmacokinetics and pharmacodynamics as secondary outcomes. Eplontersen was well-tolerated, with no serious adverse events reported. Unlike patisiran and inotersen, no local injection site reactions or renal and hematological abnormalities were noted. Eplontersen exhibited rapid absorption and dose-dependent reductions in TTR levels compared to placebo [38]. The NEURO-TTRansform trial (NCT01737398), an open-label, single-group, phase 3 study, investigated Eplontersen in adults with ATTRv polyneuropathy, NIS 10-130, and a documented TTR variant [13]. Eplontersen treatment significantly reduced serum transthyretin levels and demonstrated significant improvement in both the mNIS+7 composite score and Norfolk QoL-DN compared to placebo [13]. Adverse events led to study drug discontinuation by week 66 in a small percentage of patients (4%) in the eplontersen group versus 3% in the placebo group [14]. In the cardiomyopathy subgroup, eplontersen treatment was associated with improvements in left ventricular ejection fraction (LVEF) of 4.3% (95% CI 1.40-21.01; p-value = 0.049) and stroke volume 10.64 mL (95% CI 3.99-17.29; p-value = 0.002) [39, 40]. A retrospective study by Yu AL et al. also indicated the potential efficacy of eplontersen in managing hATTR-CM [41]. A phase III multicenter, double-blind study is currently underway to further evaluate the efficacy of eplontersen in patients with ATTR-CM [41]. The study's primary endpoint is cardiovascular mortality and cardiovascular events at week 140 and is expected to be completed in November 2025 [42].

Future perspectives for RNAi-based treatments for ATTR

New clinical trials are exploring gene-silencing agents that target mRNA to lower TTR protein levels, demonstrating the cardiac benefits of long-term treatment [43]. NTLA-2001, a CRISPR-based gene-editing therapy, and Acoramidis, a selective TTR stabilizer, show significant potential in treating ATTR amyloidosis [44, 45]. NTLA-2001 aims to reduce serum TTR concentration by knocking out the TTR gene using a lipid nanoparticle encapsulating Cas9 protein mRNA and a single guide RNA [44]. In a recent clinical trial (NCT04601051), NTLA-2001 (Intellia/Regeneron) administration led to lasting TTR knockout post single dose with minimal adverse events [44]. However, off-target effects remain a potential concern with CRISPR technology, addressed through a rigorous process of guide RNA selection targeting the specificity of the TTR gene [45]. Acoramidis (AG10), a selective, oral TTR stabilizer, is under development for transthyretin amyloidosis cardiomyopathy (ATTR-CM) [46]. In a recent phase 3 trial, Acoramidis outperformed the placebo in treating transthyretin amyloid cardiomyopathy, suggesting it is a promising treatment option [47].

While TTR tetramer stabilizers, silencers, and gene-editing therapies aim to prevent ATTR accumulation, they do not directly address existing amyloid deposits in the heart [48]. A novel treatment approach involves antibody-mediated removal of amyloid [48]. NI006, a recombinant human monoclonal IgG1 antibody, induces antibody-mediated phagocytosis of ATTR fibrils, removing ATTR deposits from tissues [48]. In a first-in-human, double-blind, placebo-controlled trial of NI006 (NCT04360434) for amyloid transthyretin cardiomyopathy, reduced cardiac tracer uptake and extracellular volume on MRI were indicative of decreased cardiac amyloid load over 12 months [49]. Molecules like TabFH2 inhibit TTR aggregation catalyzed by preformed amyloid fibrils, potentially halting disease progression in patients undergoing organ transplantation when combined with other therapies like tafamidis or Patisiran [50]. Combining TTR stabilizers, gene silencing therapies, and anti-seeding inhibitors may represent a novel approach.

## Conclusions

The introduction of RNA interference therapeutics has revolutionized the approach to managing ATTR, shifting from merely symptomatic management to intervening in the pathophysiology of the disease process. These new FDA-approved drugs for ATTR, known for their reasonable safety profile and efficacy, expand the boundaries of ATTR management. Patisiran showed promising results, with less worsening of mNIS+7 at 18 months, less decline in quality-of-life indices, and improvements in other secondary outcomes like the Norfolk QoL-DN and COMPASS-31 scale in phase 3. While Inotersen demonstrated significant improvement in neurological function and quality of life measures in Phase II and III, and sustained the benefit after two years of treatment in Phase IV, the major safety concerns are glomerulonephritis and thrombocytopenia, requiring routine monitoring of renal function and platelets. Eplontersen, on the other hand, is well tolerated with no serious adverse effects reported. It showed a substantial reduction in serum transthyretin and significant improvements in the mNIS+7 composite score and Norfolk QoL-DN. Additionally, it showed an improvement in LVEF in the cardiomyopathy group, but cardiovascular events and mortality have yet to be studied. Vutrisiran, which acts similarly, has an acceptable safety profile with significant improvement in mNIS+7 at nine months. The future of RNAi, in conjunction with the rapidly growing field of lipid nano vehicles, will serve as a new aid for ATTR. Clinical trial results, especially for amyloid cardiomyopathy, are promising. Follow-up studies with large populations and extended durations are required to understand any other adverse effects, disease recurrence, effects on mortality indices, etc.
